# Data of the Insect Biome Atlas: a metabarcoding survey of the terrestrial arthropods of Sweden and Madagascar

**DOI:** 10.1038/s41597-025-05151-0

**Published:** 2025-05-21

**Authors:** A. Miraldo, J. Sundh, E. Iwaszkiewicz-Eggebrecht, M. Buczek, R. Goodsell, H. Johansson, B. L. Fisher, D. Raharinjanahary, E. T. Rajoelison, C. Ranaivo, C. Randrianandrasana, J.-J. Rafanomezantsoa, L. Manoharan, E. Granqvist, L. J. A. van Dijk, L. Alberg, D. Åhlén, M. Aspebo, S. Åström, A. Bellviken, P.-E. Bergman, S. Björklund, M. P. Björkman, J. Deng, L. Desborough, E. Dolff, A. Eliasson, H. Elmquist, H. Emanuelsson, R. Erixon, L. Fahlen, C. Frogner, P. Fürst, A. Grabs, H. Grudd, D. Guasconi, M. Gunnarsson, S. Häggqvist, A. Hed, E. Hörnström, H. Johansson, A. Jönsson, S. Kanerot, A. Karlsson, D. Karlsson, M. Klinth, T. Kraft, R. Lahti, M. Larsson, H. Lernefalk, Y. Lestander, L.-T. Lindholm, M. Lindholm, U. Ljung, K. Ljung, J. Lundberg, E. Lundin, M. Malmenius, D. Marquina, J. Martinelli, L. Mertz, J. Nilsson, A. Patchett, N. Persson, J. Persson, M. Prus-Frankowska, E. Regazzoni, K.-G. Rosander, M. Rydgård, C. Sandblom, J. Skord, T. Stålhandske, F. Svensson, S. Szpryngiel, K. Tajani, M. Tyboni, C. Ugarph, L. Vestermark, J. Vilhelmsson, N. Wahlgren, A. Wass, P. Wetterstrand, P. Łukasik, A. J. M. Tack, A. F. Andersson, T. Roslin, F. Ronquist

**Affiliations:** 1 Department of Bioinformatics and Genetics, Swedish Museum of Natural History, Box 50007, SE 10405 Stockholm, Sweden; 2 Department of Biochemistry and Biophysics, National Bioinformatics Infrastructure Sweden, Science for Life Laboratory, Stockholm University, Box 1031, SE 17121 Solna, Sweden; 3 Institute of Environmental Sciences, Faculty of Biology, Jagiellonian University, ul. Gronostajowa 7, PL 30387 Kraków, Poland; 4 Station Linné, Ölands Skogsby 161, SE 38693 Färjestaden, Sweden; 5 California Academy of Sciences, San Francisco, CA 94118 USA; 6 Madagascar Biodiversity Center, Antananarivo, 101 Madagascar; 7 National Bioinformatics Infrastructure Sweden (NBIS), SciLifeLab, Department of Laboratory Medicine, Lund University, Lund, Sweden; 8 Department of Ecology, Environment and Plant Sciences, Stockholm University, SE 10691 Stockholm, Sweden; 9 Department of Biological and Environmental Sciences, University of Gothenburg, Box 463, SE 40530 Gothenburg, Sweden; 10 Gothenburg Global Biodiversity Centre, University of Gothenburg, Box 463, SE 40530 Gothenburg, Sweden; 11 University of Skövde, Högskolevägen 1, SE 54128 Skövde, Sweden; 12 Swedish Polar Research Secretariat, Abisko Scientific Research Station, Vetenskapensväg 38, SE 98107 Abisko, Sweden; 13 Department of Physical Geography, Stockholm University, Svante Arrhenius väg 8, SE 10691 Stockholm, Sweden; 14 Tornedalens Folkhögskola, Matarengivägen 24 G, SE 95731 Övertorneå, Sweden; 15 Department of Botany, Swedish Museum of Natural History, PO Box 5007, SE 10405 Stockholm, Sweden; 16 Department of Earth Sciences, University of Gothenburg, SE 40530 Gothenburg, Sweden; 17 Länsstyrelsen Jämtland, SE 83186 Östersund, Sweden; 18 Science for Life Laboratory, Department of Gene Technology, KTH Royal Institute of Technology, Stockholm, Sweden; 19 Department of Ecology, Swedish University of Agricultural Sciences (SLU), Ulls väg 18B, Uppsala, 75651 Sweden; 20 Organismal and Evolutionary Biology Research Programme, Faculty of Biological and Environmental Sciences, FI-00014 University of Helsinki, Helsinki, Finland; 21 Present Address: AllGenetics & Biology SL., Cubelos 21 bajo A2, Perillo, Oleiros 15172 Spain; 22 Present Address: BaSS, Biodiversity and Sustainability Solutions, Rua da Liberdade 75, 2050-023 Aveiras de Baixo, Portugal

**Keywords:** Biodiversity, Community ecology

## Abstract

We present the data from the Insect Biome Atlas project (IBA), characterizing the terrestrial arthropod faunas of Sweden and Madagascar. Over 12 months, Malaise trap samples were collected weekly (biweekly or monthly in the winter, when feasible) at 203 locations within 100 sites in Sweden and weekly at 50 locations within 33 sites in Madagascar; this was complemented by soil and litter samples from each site. The field samples comprise 4,749 Malaise trap, 192 soil and 192 litter samples from Sweden and 2,566 Malaise trap and 190 litter samples from Madagascar. Samples were processed using mild lysis or homogenization, followed by DNA metabarcoding of CO1 (418 bp). The data comprise 698,378 non-chimeric sequence variants from Sweden and 687,866 from Madagascar, representing 33,989 (33,046 Arthropoda) and 77,599 (77,380 Arthropoda) operational taxonomic units, respectively. These are the most comprehensive data presented on these faunas so far, allowing unique analyses of the size, composition, spatial turnover and seasonal dynamics of the sampled communities. They also provide an invaluable baseline against which to gauge future changes.

## Background & Summary

Insects comprise the vast majority of macroscopic biodiversity^1^ and are crucial for ecosystem functioning^2–5^. Yet, despite their ecological importance and remarkable diversity, insects remain one of the least understood organism groups. An estimated 70–85% of insect species still remain unknown to science^6,7^ and for the large majority of those described, we know little about their biology, distribution, or population trends^8^. Recent studies have indicated a decline in terrestrial insect biomass and diversity during the last decades^9–16^, but the data are often limited in taxonomic, geographic and/or temporal scope, hindering comprehensive conclusions to be reached^17–21^. It is clear that existing data on insect biodiversity provide only fragmented insights into how many insect species there are, how they are structured into communities and how these communities contribute to healthy ecosystems.

Comprehensive longitudinal sampling across and within regions is urgently needed to close this knowledge gap. However, setting up such long-term monitoring projects faces many challenges, from financial constraints, complex management and coordination hurdles to methodological limitations in the processing and identification of collected material. Indeed, a significant barrier has been the absence of cost-effective methods for the comprehensive inventorying of insect faunas. For example, one of the most ambitious countrywide insect inventory projects to date, the Swedish Malaise Trap Project (SMTP), collected an estimated 20 million specimens at 50 sites over a three-year period^22^. Sorting the material into 350 taxonomic fractions suitable for specialist processing took 15 years. During this time period, only 0.85% of the specimens had been identified to species, despite a substantial effort from numerous taxonomists^22,23^.

Fortunately, in recent years, the traditional insect inventorying methods relying on morphological identification by taxonomic experts have been complemented by considerably faster and more cost-efficient methods for processing insect samples and delineating species proxies, relying on DNA metabarcoding. This has revolutionized our capacity to comprehensively study and effectively monitor insect faunas^24^.

In the Insect Biome Atlas (www.insectbiomeatlas.org) project, we used DNA metabarcoding of an extensive set of community samples to characterize the terrestrial arthropod faunas of Sweden and Madagascar, and their associated microbiomes, in unprecedented detail. Sweden and Madagascar were chosen because they represent two countries of approximately the same size but with completely different biogeographic and geological history. The Swedish fauna has been assembled by independent migration from different refugia after the last glacial maximum, some 20,000 years ago^25^. In contrast, the Malagasy fauna has largely evolved in place, and in isolation^26^. The environmental factors also contrast sharply: Sweden is characterized by a northern temperate climate and an elevational gradient running mainly from the east coast to the Scandinavian mountains in the west, whereas Madagascar is marked by a tropical climate and a significantly more complex topographical setting. Thus, any similarities between Sweden and Madagascar clearly represent non-random structuring of the faunas, while the differences between them illustrate much of the range of global variation one might expect among faunas.

**Fig. 1:**
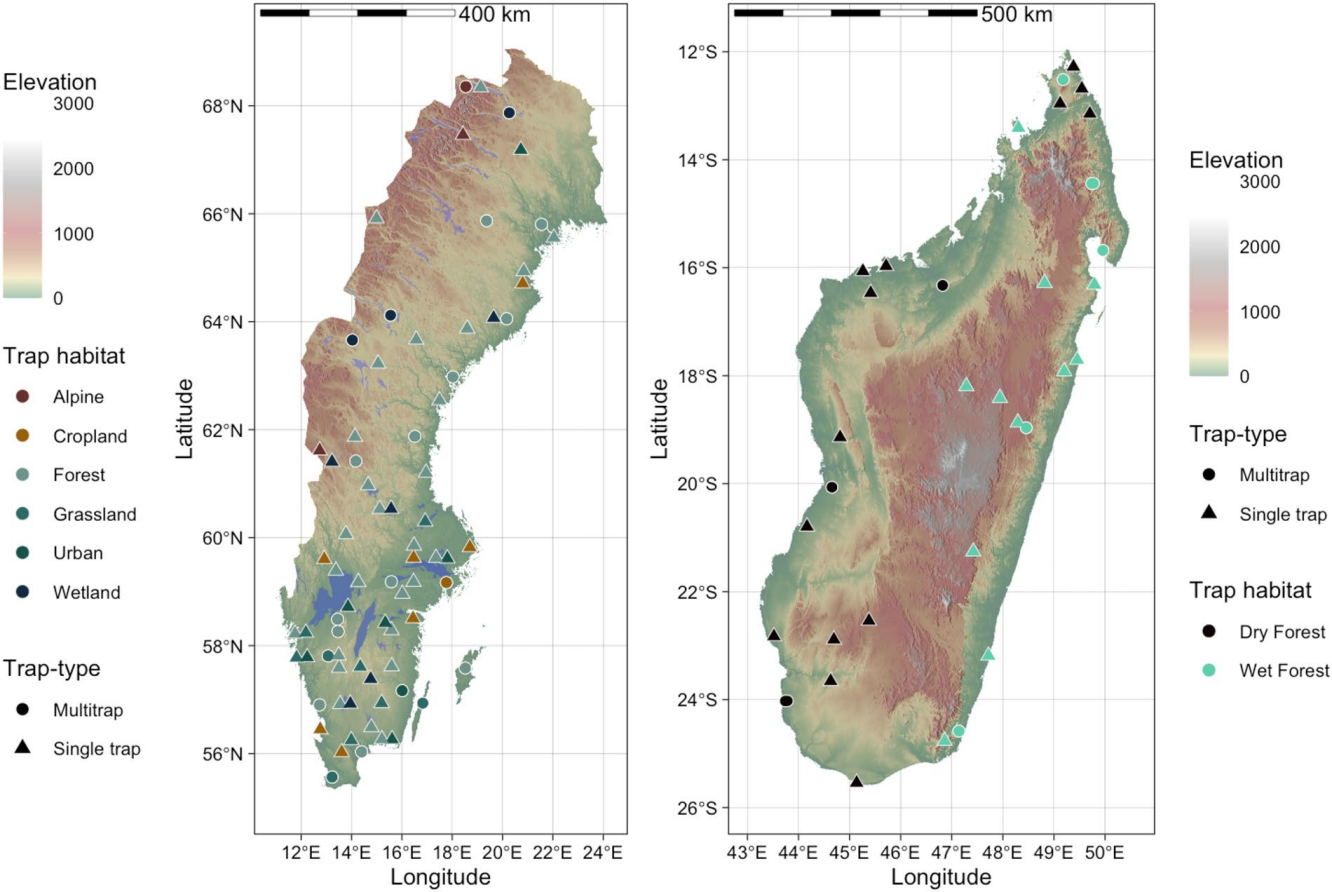
Sampling sites for Sweden (left) and Madagascar (right). Sampling sites with multiple Malaise traps (multitrap sites) are depicted with filled circles and single trap sites with filled triangles. Colours of each point denote the habitat the trap was placed in. In Sweden, a total of 203 Malaise traps were placed across 100 sampling sites: 102 traps placed in forests, 33 in croplands, 26 in wetlands,16 in grasslands, 14 in alpine and 12 in urban areas for Sweden. For multitrap sites only the main habitat sampled is shown (to see all habitat sampled at these locations please check ´sites_metadata´ file available at Figshare^60^. In Madagascar, sampling sites were associated with forested habitats located within protected areas only. Fifty Malaise traps across 33 sites were deployed: 27 Malaise traps in rainforests (16 sites, denoted in white) and 23 Malaise traps in dry forests (17 sites, denoted in black). Background map colour illustrates elevation.

Over a period of 12 months in 2019-20, Malaise trap samples were collected weekly (spring to autumn) or biweekly to monthly (winter, when feasible) at 100 sites (203 locations) in Sweden and weekly at 33 sites (50 locations) in Madagascar (Fig. 1). Sites and trap locations were selected to provide a representative sample of Swedish habitat types. In brief, sites were stratified across the habitats in rough proportion to the relative coverage of habitats in the Swedish landscape^27^. In Madagascar, only forests (National Parks) were targeted due to logistical constraints. A hierarchical design involving both single-trap and multitrap sites was used to allow more powerful analyses of spatial turnover. The Malaise traps were maintained by citizen science volunteers in Sweden and park rangers in Madagascar. This approach not only substantially mitigated sampling-related challenges but also engaged a diverse range of stakeholders in the scientific process.

To further characterize soil arthropods and soil microbiomes, we complemented the Malaise trap samples with soil and litter samples. In addition, we collected environmental data and data on ecosystem functions at each site. In total, the project yielded 4,753 Malaise trap samples, 188 soil samples and 189 leaf litter samples from Sweden and 2,566 Malaise trap samples and 190 leaf litter samples from Madagascar.

Samples were processed using mild lysis or homogenization, followed by DNA metabarcoding of CO1 (418 bp) on the NovaSeq 6000 platform using optimized protocols developed in the project^28–30^. A novel pipeline for processing the metabarcoding data - developed in the project - was used for quality filtering and taxonomic characterization of the samples based on these data^31^. This pipeline utilizes the extensive amounts of samples collected in the project to provide data of significantly improved quality and accuracy compared to the current state of the art.

Here we present the key datasets resulting from the project. The data on ecosystem functioning and arthropod wet biomass have been published elsewhere^32,33^ and the data on soil microbiome (bacterial 16S and fungal ITS2) and microbiome associated with the arthropod communities in Sweden will be presented separately.

The CO1 data presented here comprise 698,378 non-chimeric sequence variants from Sweden and 687,866 from Madagascar, representing 33,989 and 77,599 operational taxonomic units (OTUs), respectively, after filtering and cleaning. The Swedish OTUs were distributed across 11 phyla, 26 classes and 97 orders. Of the OTUs, 79%, 50%, 34% and 60% were classified at the level of Family, Genus, Species and BOLD BIN^34^, respectively. The low annotation success at the species level compared to the BOLD BIN level is partly due to name resolution problems for BOLD BINs—such as several synonyms being in active use, or the same species being placed in different genera by different annotators—and partly due to failures of CO1 barcodes in accurately circumscribing closely related species. Distinguishing between these cases would require detailed analysis on a case-by-case basis. For Madagascar, the OTUs were distributed across 9 phyla, 21 classes and 90 orders, with 28%, 4%, 2% and 9% of OTUs classified at the level of Family, Genus, Species and BOLD BIN, respectively. For both datasets, the phylum Arthropoda accounted for ≥99.5% of total reads and >97% of OTUs. Calibration curves relating wet biomass to the number of specimens suggest that the analyzed Malaise trap samples comprise around 7.0 million insects from Sweden and 1.7 million insects from Madagascar. However, our abundance estimates for Madagascar rely on a biomass-count extrapolation from Swedish data, which may be less accurate if the average biomass per individual differs substantially between these two regions.

The datasets we present here offer profound insights into the diversity, composition and structure of the terrestrial arthropod communities of Sweden and Madagascar. They also provide an invaluable baseline against which to gauge future changes in these faunas. To maximize the utility of this baseline, new studies should preferably use the same lab protocols and rerun the bioinformatic processing with new and old data combined. Eventually, more complete reference libraries are likely to improve the taxonomic annotation of our dataset to the extent that it will provide a useful baseline for future studies regardless of their methodology. While the Insect Biome Atlas project focuses exclusively on Sweden and Madagascar, we expect that it will serve as a template for similar studies in other regions. We also hope that it encourages a wider interest in comprehensive studies of insect faunas and in establishing long-term monitoring of insect populations globally.

## Methods

### Site selection

In Sweden, sampling sites were selected following a stratified design based on the major landscape types as identified by the National Inventory of Landscapes in Sweden (NILS)^27^, for which extensive data on land cover, land use and landscape structure spanning 18 years (2003–2020) are available (https://landskap.slu.se/nils/dv). Malaise traps were set up within a radius of 5 km from the closest NILS sampling plot. A total of 203 Malaise traps were deployed within six major habitats across 100 sites: 102 traps in forests, 33 in croplands, 26 in wetlands,16 in grasslands, 14 in alpine and 12 in urban areas (Fig. 1). The proportion of Malaise traps by habitat is representative of the contribution of each habitat type to the overall Swedish landscape (Table 1), with down-weighting of the most common habitat (forests, which account for 60% of the landscape according to NILS) and some up-weighing of the remaining, sparser habitats (croplands and wetlands, which account for 8% of the landscape each; and urban sites and grasslands, which each account for 3% of the landscape). At 21 of the 100 sampling sites, we implemented a hierarchical design by deploying up to 6 Malaise traps instead of one. In Sweden there were 12 multi-trap sites with between 2 and 6 traps, and in Madagascar there were 9 multi-trap sites with between 2–4 traps. Distances between Malaise traps at these multitrap sites ranged from 90 to 1200 meters, sampling two different habitats when possible (three Malaise traps per habitat). In Madagascar, sampling sites were associated with forested habitats located within protected areas only. In total we deployed 50 Malaise traps across 33 sites: 27 Malaise traps in rainforests (16 sites) and 23 Malaise traps in dry forests (17 sites) (Fig. 1). At eight of the 33 sites, we implemented a hierarchical design by deploying 3 traps within a single habitat. Distances between Malaise traps at these multitrap sites ranged from 100 to 4700 meters. Malaise traps were set up by a team of fieldworkers together with the trap managers at each site: 150 volunteers in Sweden and 107 local community members and park rangers in Madagascar. At the time of setting up the Malaise traps, trap managers were trained on how to set up and maintain the trap, retrieve the samples from the trap and collect metadata associated with the sampling event.

**Table 1:** Number (n) and proportional representation (%) of Malaise traps per habitat and area (m2) and proportion (%) of each habitat in Sweden.

Habitat	Area		Malaise traps	
	m2	%	n	%
alpine	170195	8	14	7
aquatic	205368	9	0	0
croplands	163551	8	33	16
forest	1294787	60	102	50
grasslands	64772	3	16	8
substrate	14430	1	0	0
urban	74943	3	12	6
wetlands	177123	8	26	13
Total	2165170	100	203	100

Data on area of each habitat was retrieved from the National Inventory of Landscapes in Sweden (NILS) 27. Note that area presented here does not correspond to the total area of habitats present in Sweden but only to the sampled habitat plots in NILS. Habitats “substrate” and “aquatic” were not sampled in this project as they were not suited for Malaise trap nor soil or litter sampling.

### Standing characteristics and other abiotic data

At each Malaise trap location in Madagascar, we measured a set of standing characteristics. To assess tree density, we set up four 10 m long transects, one on each side of the trap, and measured the diameter at breast height (DBH) of all trees with its roots or stem within 10 cm of the transect. For multi-stemmed trees, we measured only the DBH of the largest stem. We included measurements for trees, lianas (L), palms (P) and ferns (F). We also measured the circumference of all trees with a DBH larger than 30 cm within a 10 m radius of the Malaise trap. Finally, we measured canopy cover at each Malaise trap location by taking five photographs of the canopy at 2 meters height straight up to the canopy. One photograph was taken at the center of the Malaise trap and the other four photographs were taken five meters away from the trap, one on each side of the trap. Each photograph was analysed with ImageJ software to get a percentage of canopy cover.

At each Malaise trap location for both Sweden and Madagascar we also measured soil nutrients and soil pH. Topsoil (0–20 cm) was sampled at 5 sites around each Malaise trap: one soil core (6 cm diameter) at the center of the trap and one soil core on each of the four “sides” of the trap, five meters away from the trap. Soil samples were taken as composite samples from the five locations. At each site, prior to taking the soil cores, we measured leaf litter depth using a ruler and soil humidity with the SM150 soil moisture kit, Delta-T, United Kingdom. Soil samples from Sweden were sent to Eurofins to measure the following macronutrients: AL-extractable P, K, Mg and Ca; NH_4_; NO_3_; K/Mg ratio and mineral N (Nmin). The concentrations of soil nutrients are presented as mg·100 g^−1^ air-dry sieved soil (<2mm). Soil samples from Madagascar were sent to the Laboratoire des Radioisotopes, Madagascar, to measure the following macronutrients: organic C (g/Kg), total N (g/Kg), total P (g/Kg), exchangeable K (cmol/Kg), exchangeable Ca (cmol/Kg) and exchangeable Mg (cmol/Kg).

### Sampling for biotic characterization

#### Malaise trap samples

Arthropods were collected in individually barcoded bottles pre-filled with 400 mL of 95% ethanol attached to each Malaise trap. To facilitate the recording of metadata associated with each sampling event, trap managers used a mobile application specifically designed for the project. The app allowed scanning the individually barcoded Malaise trap and sample bottle at the time of collection so that each sampling event was automatically associated with a specific trap. When the barcode of each sample was scanned, the app automatically registered the GPS coordinates and the date and time of the collection event in addition to any other metadata manually inserted by the user, such as the trap condition at collection. In Sweden, Malaise traps were active between January and December 2019. Samples were collected every week during spring to autumn (March/April to September/October depending on latitude) and monthly or bi-weekly in the winter (October/November to March/April, depending on latitude). In the northern part of the country, we did not collect at all during the winter months, when snow and strong winds prevented proper operation of the traps. The sampling strategy was based on previous experiences from the Swedish Malaise Trap Project^22^, and was deemed to result in minimal loss of sampled specimens and an acceptable loss of phenological resolution. The effort resulted in 4,753 insect community samples from Sweden. In Madagascar, Malaise traps were active between August 2019 and July 2020. Samples were collected every week throughout that period, resulting in 2,566 insect community samples. Every fourth sample from each trap location (638 samples in total, roughly one sample per month), was left at the Madagascar Biodiversity Center (https://www.madagascarbio.org/) to help build a natural history collection of Malagasy insects in the country of origin. The remaining samples were shipped to Sweden for DNA extraction and metabarcoding as described below.

#### Soil and litter samples

Soil and leaf litter arthropod communities were sampled during the growing season (26th of June 2019 to 27th of July 2019) at each Malaise trap location in Sweden. Leaf litter arthropod communities were sampled by collecting a total volume of 1 L of leaf litter from five different sites around each Malaise trap, 2.5 meters away from the nearest extremity of the trap. Soil arthropod communities were sampled using soil cores (5.5 cm diameter) with a depth of 10 cm at exactly the same five sites where litter samples were taken. The five soil core samples from each Malaise trap location were pooled and mixed by hand immediately after collection, then stored in a cool place. Within 48 h after collection, living arthropods were extracted from soil and litter samples over a period of 96 hours using Berlese funnel^35^. In brief, arthropods were extracted by placing the soil samples in metal net baskets (1.5 mm mesh) in the top of stainless metal funnels (diam. 18 cm). Twenty funnels were placed together on a wooden board with holes for the funnels. Approximately, 17 cm above the funnels, a heating plate (max 950 watt, starting at 25% and gradually increasing to 75%) were switched on to create a heat gradient and LED-lamps were switched on to provide light. To cause the arthropods to exit the substrate before being trapped in the dried-out structure, we gradually increased the temperature over the first 24 hours to a temperature of 52 °C. This temperature was then held constant for the remaining 72 hours. Arthropods were collected directly into 100 mL plastic bottles filled with 95% ethanol. Leaf litter arthropod communities were also sampled at each Malaise trap location in Madagascar. Here we collected four samples in each direction of the Malaise trap (back, front, left and right), five meters away from it. Leaf litter sampling involved sifting and concentrating 2 L of leaf litter from a 1 m^2^ square using a Winkler-sifter. Before sifting, the leaf litter was minced with a machete to dislodge any insects hiding in the twigs or decayed logs. After sifting, the leaf litter was stored in a cloth bag before extracting the arthropods. Within 12 hours of sampling, living arthropods were extracted at ambient temperature overnight into a 100 mL bottle filled with 95% ethanol using a mini-Winkler extractor for a total of 12 hours^36^.

### DNA extraction

#### Malaise trap samples

**Fig. 2:**
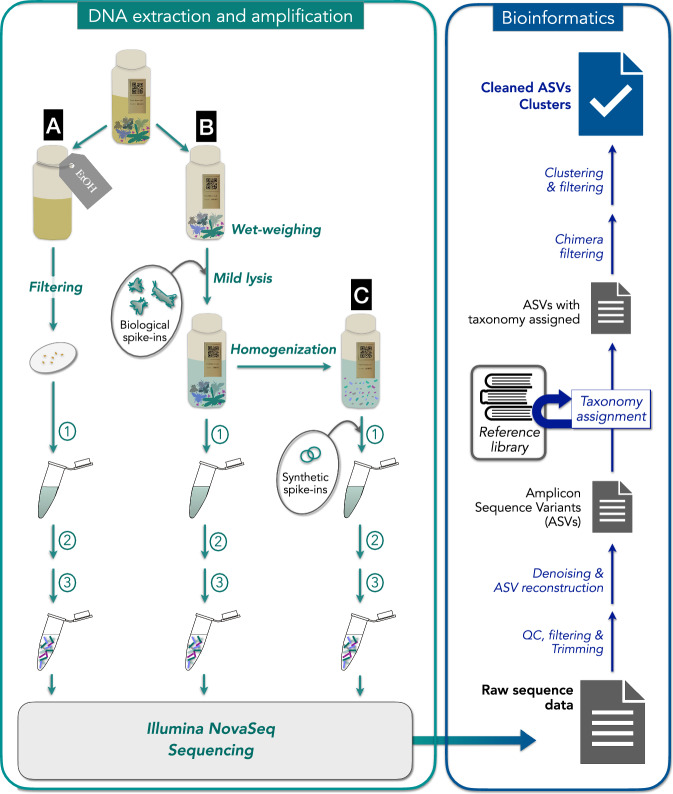
Schematic representation of sample processing and bioinformatic pipeline. Malaise trap sample processing in the lab (left panel). DNA was extracted from (**A**) the ethanol filtered from malaise trap samples; (**B**) the lysates using the FAVIS mild lysis protocol^29^; and (**C**) the homogenates^30^. Before lysis, biological spike-ins and synthetic spikes were added to the samples in B and C, respectively. After lysis, DNA was purified using silica-coated magnetic beads with the KingFisher Cell and Tissue DNA kit on a KingFisher Flex 96 robot (1). After DNA purification, a 418 bp fragment of the mtDNA cytochrome c oxidase subunit 1 (CO1) gene was amplified using a 2-step PCR approach. In the first step (2), the target region was amplified using broad-spectrum primers BF3 CCHGAYATRGCHTTYCCHCG^42^ and BR2 CDGGRTGNCCRAARAAYCA^43^. In the second step (3), indexes were added and Illumina adapters completed. Samples were pooled and library pools were sequenced on a NovaSeq 6000 instrument using the ‘NovaSeqXp’ workflow in ‘SP 500-cycle’ flow cells, with 384 double-uniquely indexed samples per lane. Schematic representation of processing sequence data from raw reads to ASV clusters (right panel). Briefly, paired end reads were trimmed in a series of steps using the program cutadapt^47^ and filtered to retain only sequences that were between 403 and 418 nt in length (in incremental steps of 3 nt) and that did not contain any in-frame stop codons. Preprocessed reads were then denoised using DADA2^49^ to infer amplicon sequence variants (ASVs). The ASVs were then processed using the HAPP pipeline^31^ to taxonomically annotate the ASVs (using SINTAX classifier and a purposely built CO1 database). Taxonomic assignments obtained from SINTAX were refined using phylogenetic methods with EPA-NG^55^ into an insect phylogeny^56^, followed by taxonomic assignment with gappa^57^. ASVs were then clustered into OTUs using Swarm^59^. Finally, the clustered data was cleaned from NUMTs and other types of noise using the NEEAT algorithm^31^.

DNA was extracted from Malaise trap samples using both non-destructive (mild lysis and preservative ethanol) and destructive methods (homogenization) (Fig. 2).

1. Mild lysis: DNA was extracted from 6,483 Malaise trap samples (4,560 from Sweden and 1,923 from Madagascar) using a non-destructive mild-lysis protocol (FAVIS protocol, steps 1-17^29^). In brief, ethanol was first decanted from each sample and insect wet biomass was measured. Lysis buffer, proteinase K and biological spike-ins (specimens from foreign species, i.e., species that don’t occur in the respective countries) were added and samples were incubated for 2h45m at 56 °C in a dry shaking incubator. After the incubation period the lysate was drained and each insect community was remixed with the previously-decanted ethanol for long-term storage. DNA was purified from 225 μL of lysate using silica-coated magnetic beads with the KingFisher Cell and Tissue DNA kit on a KingFisher Flex 96 robot (both Thermo Scientific, ThermoFisher Inc, United States of America) according to manufacturer instructions. After DNA purification, DNA extracts from 12 samples in each 96-well plate were quantified using Qubit™ dsDNA HS Assay Kit on a Qubit Fluorometer (Invitrogen™, Thermo Scientific, ThermoFisher Inc, United States of America). DNA concentration of those samples is available as Supplementary Table S1. The list and number of biological spike-ins added in each country (SE and MG) can be retrieved at from Figshare^37^.
2. Homogenization: Approximately every fourth sample collected at each sampling site in Sweden (n = 873) was further processed using a destructive homogenization protocol^30^ after mild lysis. In brief, after decanting preservative ethanol we homogenized each bulk sample into an “arthropod soup” using the ULTRA-TURRAX® Tube Drive P, IKA®-Werke GmBH & Co. KG, Germany. For the homogenization we used single use DT-50 Dispersing tubes. After homogenization, the entire “arthropod soup” was digested with lysis buffer and proteinase K for 2h45min at 56 °C in a dry shaking incubator. To make sure that we used all available DNA from each sample, we combined the homogenate with the respective lysate obtained during mild lysis before proceeding with DNA purification. At this time we also added a standardized amount (5 million copies) of two synthetic oligonucleotide sequences (synthetic spike-ins) to each homogenate aliquot. Synthetic spike-ins were produced as described in Iwaszkiewicz-Eggebrecht *et al*.^38^ and their sequences can be found at Figshare^37^. DNA was purified from a 225 µL subsample of homogenate using silica-coated magnetic beads with the KingFisher Cell and Tissue DNA kit on a KingFisher Flex 96 robot (both Thermo Scientific, ThermoFisher Inc, United States of America) according to manufacturer instructions. After DNA purification, DNA extracts from 12 samples (including positive and negative controls) in each 96-well plate were quantified using Qubit™ dsDNA HS Assay Kit on a Qubit Fluorometer (Invitrogen™, Thermo Scientific, ThermoFisher Inc, United States of America). DNA concentration of those samples is available as supplementary material (Dataset 1).
3. Preservative ethanol: For 15 Malaise trap samples we also extracted DNA from the preservative ethanol (prior to mild lysis and homogenization). Ethanol was first decanted from each sample as described in step 7 of the FAVIS protocol^29^. The decanted ethanol was then manually filtered using a Millipore® Sterivex™ filter unit (pore size of 0.22 μm) (Merck KGaA, Germany). After filtration, DNA was lysed inside the Sterivex™ unit by adding 540 µl of lysis buffer (ATL, Qiagen, Germany) and 60 µl proteinase K (Qiagen, Germany) and incubating the unit at 56 °C overnight. DNA was transferred from the Sterivex™ unit into a DNeasy® Blood and Tissue Kit column (Qiagen, Germany) for purification, following manufacturer instructions.

#### Soil and litter samples

Arthropod litter samples from Madagascar were processed using the same mild lysis protocol used for the Malaise samples described above, with the exception of not adding biological spike-ins to the samples. For Sweden, we used a 1 mm wire-sieve to separate each sample (from soil or litter) into two components based on size: the macrofauna subsample, composed mainly of adult and larval Coleoptera, and the mesofauna subsample, dominated by mites and springtails. To remove debris and dirt accumulated in the mesofauna subsamples, we processed them further using a combination of flotation in distilled water, adapted from the Flotation-Berlese-Flotation protocol in^39,40^, followed by vacuum pump filtration (41 µm nylon filter). DNA was extracted separately from the macrofauna and the mesofauna subsamples using the Thermo Scientific KingFisher Cell and Tissue DNA Kit, ThermoFisher Inc, United States of America. Ethanol was first dried from each subsample in a dry incubator at 40 °C for 4-5 hours. After drying, specimens were manually homogenized with a pestle in a 50 mL single use falcon tube and lysed overnight at 56 °C by adding 800 µL of lysis buffer and 100 µL of proteinase K (provided in the Kingfisher Cell and Tissue DNA kit). After each use pestels were sterilized by washing in a 10% bleach bath for 15 minutes, followed by rinsing with water. DNA was then purified from a 225 µL aliquot of homogenate using silica-coated magnetic beads with the KingFisher Cell and Tissue DNA kit on a KingFisher Flex 96 robot (both Thermo Scientific, ThermoFisher Inc, United States of America) following manufacturer instructions. DNA extracts of all samples were quantified using Qubit dsDNA HS Assay Kit on a Qubit Fluorometer (Invitrogen™, Thermo Scientific, ThermoFisher Inc, United States of America), and the original sample was reassembled by combining DNA extracts from each subsample pair in a ratio of 1:10 (amount of DNA of macrofauna: amount of DNA of mesofauna). This minimizes the bias in the sequencing depth due to biomass differences between the two components of each sample^39,40^.

### Library preparation and sequencing

To characterize arthropod communities, we amplified a 418 bp fragment of the mtDNA cytochrome *c* oxidase subunit 1 (CO1) gene, using the purified DNA extracted from the Malaise trap samples (lysates, homogenates and preservative ethanol) and soil and litter samples. Each bulk sample was metabarcoded using a two-step PCR approach for library preparation (method 4 of^41^). In the first step (PCR 1), the target region was amplified using broad-spectrum primers BF3 *CCHGAYATRGCHTTYCCHCG*^42^ and BR2 *CDGGRTGNCCRAARAAYCA*^43^. The primers were supplemented with 5′-end Illumina sequence adapters (forward: ACACTCTTTCCCTACACGACGCTCTTCCGATCT-3′, reverse: 5′-GTGACTGGAGTTCAGACGTGTGCTCTTCCGATCT). To increase the complexity of the libraries, each primer was further complemented with variable length inserts (TGA, GA, A, or no base for the forward primer, and GAT, AT, T, or no base for the reverse primer)between the adapter sequence and the target-binding region, generating phased primers in equal proportions^44,45^. PCR 1 reactions were carried out in a final reaction volume of 40 μL containing 20 μL Qiagen, Germany, Multiplex PCR Master mix, 1 μM of each primer, and 4 μL of template DNA (for mild lysis and preservative ethanol samples). As the DNA concentration for homogenates was relatively higher, the PCR reactions were carried out in a final volume of 10 μL with 1 μL of template DNA. The PCR conditions were 95 °C for 15 min, 25 cycles of 94 °C for 30 s, 50 °C for 90 s and 72 °C for 90 s, followed by a final elongation step of 72 °C for 10 min. PCR 1 products were cleaned with magnetic beads (Carboxyl-modified Sera-Mag Magnetic Speed-Beads, Hydrophobic, CYTIVA), using 2/1 (v/v) magnetic beads to sample ratio. In the second step (PCR 2, indexing PCR), indexes were added and Illumina adapters completed. The indexing primer design followed the Adapterama scheme^41,46^ with 10 bp indexes as follows: index i5 (forward): AATGATACGGCGACCACCGAGATCTACACxxxxxxxxxxACACTCTTTCCCTAC index i7 (reverse): CAAGCAGAAGACGGCATACGAGATxxxxxxxxxxGTGACTGGAGTTCAG. Libraries were double-uniquely indexed - in other words, each forward and each reverse index was used for only one library in a given sequencing lane. PCR 2 conditions were 95 °C for 15 min, 7 cycles of 94 °C for 30 s, 50 °C for 90 s and 72 °C for 90 s, followed by a final elongation step of 72 °C for 10 min. To form the sequencing pool, all samples were pooled approximately equimolar based on the intensity of the band in an agarose gel, and the resulting sample pool was then purified with the Promega ProNex® Size-Selective Purification System, using 1/1.5 (v/v) pool to magnetic beads ratio. The quality of the library pool was then checked with an Agilent DNA High Sensitivity Kit on a 2100 Bioanalyzer instrument (Agilent Technologies). Library pools were sequenced on a NovaSeq 6000 instrument using the ‘NovaSeqXp’ workflow in ‘SP 500-cycle’ flow cells, with 384 double-uniquely indexed samples per lane, or a total of 768 libraries per flow cell (8 × 96 well plates). This sequencing was performed at the Swedish National Genomics Infrastructure (NGI) at SciLifeLab (Solna, Sweden). The detailed step-by-step protocol can be found in^29^.

### Processing sequencing data

Sequences were preprocessed (read trimming and filtering) using a Snakemake workflow available at Github: https://github.com/biodiversitydata-se/amplicon-multi-cutadapt. In this workflow, paired end reads were trimmed in a series of steps using the program cutadapt^47^ (v3.1):

1. Discard all reads with the Illumina TruSeq adapters in either the 5’ or 3’ end of sequences.
2. Search for and trim primer sequences from the start of reads in R1 and R2 files using forward and reverse primers, respectively. Remove any untrimmed reads. This step is done with additional settings ‘–no-indels’ and ‘-e 0’ in order to only accept perfect matches.
3. Discard any remaining reads that still contain primer sequences.
4. Trim reads to a fixed length. This length is calculated by subtracting the length of the longest primer from the read length defined by the ‘expected_read_length’ parameter under the cutadapt: section in the config file (default value is 251).

Reads were then filtered to retain only sequences that were between 403 and 418 nt in length (in incremental steps of 3 nt) and that did not contain any in-frame stop codons. Preprocessed reads were denoised using the nf-core/ampliseq Nextflow workflow^48^ (v2.4.0) which uses the DADA2 algorithm^49^ to infer amplicon sequence variants (ASVs) from the preprocessed reads. Due to the indexing scheme used with unique dual indexes per sample it was not necessary to perform per-sample abundance filtering to correct for mistagging^50,51^.

The ASVs were then processed using the HAPP pipeline (https://github.com/insect-biome-atlas/happ) described separately^31^ to taxonomically annotate the ASVs, remove chimeras, cluster the ASVs into OTUs, and remove NUMTs and other noise from the data. Specifically, ASVs were taxonomically annotated using SINTAX^52^, as implemented in vsearch^53^, against a custom-made reference CO1 database available at Figshare^54^. This reference database was assembled from sequences in the BOLD database^34^ as follows. Firstly, nucleotide sequences and metadata linking record ids to BOLD BINs were downloaded from the GBIF Hosted Datasets (ibol_2022_01_17.zip). This was merged with taxonomic information for BOLD BINs obtained from the GBIF backbone (backbone.zip from 2022-11-23). The data was then filtered to only keep records annotated as ‘CO1-5P’ and assigned to a BOLD BIN ID. The taxonomic information was parsed in order to assign species names and resolve higher-level ranks for each BOLD BIN ID. Sequences were processed to remove gap characters and leading and trailing ‘N’s. After this, any sequences with remaining non-standard characters were removed. Sequences were then clustered at 100% identity using vsearch. This clustering was done separately for sequences assigned to each BOLD BIN ID. These steps were implemented in a Python package called coidb, available at Github https://github.com/insect-biome-atlas/coidb. Taxonomic assignments obtained from the kmer-based SINTAX classifier were refined using phylogenetic methods. Specifically, ASV sequences classified as Insecta or Collembola (class) but unclassified at order level by SINTAX were reassigned by phylogenetic placement with EPA-NG^55^ into an insect phylogeny^56^, followed by taxonomic assignment with gappa^57^. Assignments obtained this way were used to update the SINTAX taxonomy, but only at the order level, leaving child ranks with the ‘unclassified’ prefix.

Following taxonomic assignments ASVs were further processed to remove chimeras with uchime^58^ and the remaining non-chimeric sequences were clustered using Swarm^59^ (v3.1.0) with setting ‘-d 15’. To choose the best setting for Swarm we evaluated the performance of different settings by calculating precision and recall values in Sundh *et al*.^31^. In the datasets, we provide taxonomic assignments for all ASVs using several different approaches. We also provide consensus annotations for OTU clusters using an abundance-based consensus approach. For each cluster, starting from the lowest rank (BOLD BIN here), each unique taxonomic name was weighted by the sum of reads across samples and ASVs with said name. These sums were then normalized to percentages. If a single taxonomic name made up at least 80%, then that name was assigned to the cluster, including the assignments of parent ranks. If no single name reached the 80% consensus threshold, the process was iterated for the parent rank. Ranks for which no consensus could be reached were prefixed with ‘unresolved.’ followed by the name of the most resolved consensus taxonomy. Using this procedure, it is, in theory, possible that OTUs are assigned a consensus taxonomic label from an unclassified ASV, or to an ASV with an ambiguous name. However, this happens very rarely in practice. Out of the 33,989 OTUs in the Swedish dataset, only 282 (0.8%) get an unclassified species level assignment even though an ASV with a properly assigned species label is present in the same OTU. For Madagascar this happens for 0.1% (77) of the 77,599 OTUs. For each cluster, the ASV sequence with the highest median of normalized read counts across samples was selected as representatives of the cluster. Ties were broken by taking the ASV with the highest mean.

The clustered data was further cleaned from NUMTs and other types of noise using the NEEAT algorithm, which takes taxonomic annotation, correlations in occurrence across samples (‘echo signal’) and evolutionary signatures into account, as well as cluster abundance^31^. We used default settings for all parameters in the evolutionary and distributional filtering steps, and removed clusters unassigned at the order level and with less than three reads summed across each dataset. Additionally, we removed clusters present in more than 5% of blanks.

### Biomass and count data

To allow an assessment of how the wet biomass of a Malaise trap sample translates to the number of specimens, we counted all the specimens for 24 Malaise trap samples from Sweden. We complemented these data with wet biomass estimates and specimen counts for 224 Malaise trap samples from a separate Swedish Malaise trapping campaign in 2018–2019, the Swedish Insect Inventory Project (SIIP).

**Fig. 3:**
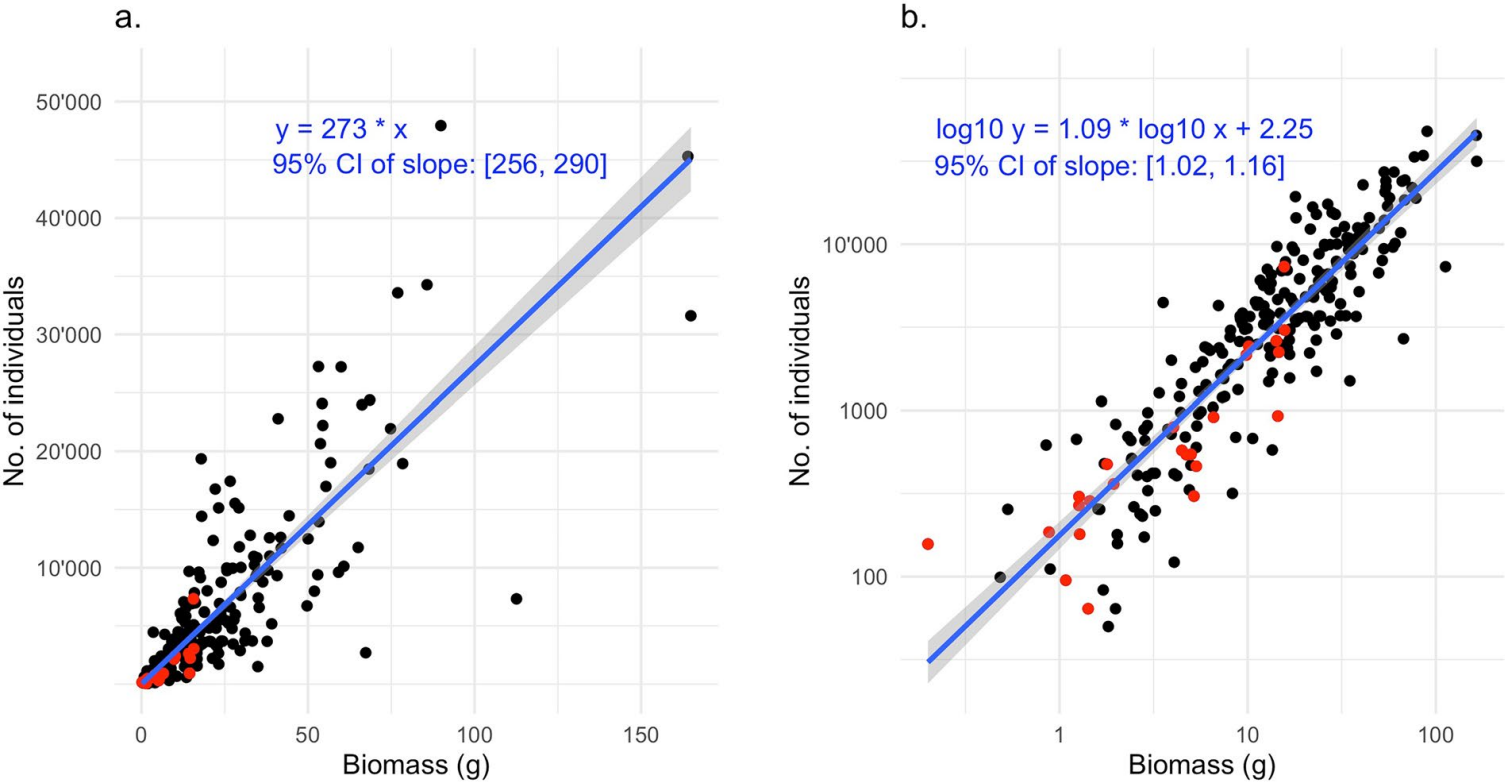
Linear regression between biomass of Malaise trap samples and the number of individuals (insect specimens). The data points are individual Malaise trap samples; the 224 black data points are from the SIIP project and the 24 red data points from the IBA project. The blue line is a fitted straight line with the 95% confidence interval marked in gray. The equations of the fitted lines are shown in blue. (**a**) The fitted line is forced through the origin, as the biomass is zero at zero individuals. The number of individuals per gram is estimated at 273 ± 17. The R-squared is 0.81. (**b**) The same dataset with logged axes. The slope is 1.09 + −0.07, that is, significantly larger than 1.0. This indicates that large samples (in terms of biomass) tend to have slightly more specimens in them than a strict proportional relationship between biomass and specimens would suggest. The R-squared is 0.79.

The data (Fig. 3) show that the number of specimens is only approximately proportional to the biomass of a sample (linear model without intercept, adjusted R^2^ 0.81; Fig. 3a). Specifically, there is a slight tendency for the larger samples (in terms of biomass) to contain more specimens than if the relation was strictly proportional, as shown in a log-log model (R^2^ 0.79, regression coefficient 1.09 ± 0.04; Fig. 3b). Fitting a linear model with intercept does not support the alternative explanation of a constant amount of alcohol residue causing this (adjusted R^2^ 0.69, intercept positive and not negative as expected under the hypothesis). Using the proportional model, the Swedish Malaise trap material is estimated to contain 7.0 M specimens, and the Madagascar material 1.7 M specimens. Accounting for the deviation from proportionality by applying the log-log regression equation to each sample separately, the Swedish material is instead estimated to contain 5.6 M specimens and the Madagascar material 1.2 M specimens.

## Data Records

The raw sequencing data (including the primer sequences) generated in this study are available at the European Nucleotide Archive (ENA) under study accession number PRJEB61109^60^. Processed sequencing data including raw ASV sequences in FASTA format and ASV count files that contain the counts of each ASV (rows) in each sample (columns) are available at Figshare^37^. These data are organized by country (Sweden/Madagascar) and contains ASVs generated from Malaise trap samples (lysates, homogenates and preservative ethanol) and soil and litter samples, together with ‘sites_metadata’, ‘samples_metadata’, ‘sequencing_metadata’ and ‘spike_ins_metadata’ files. The same repository also contains datasets describing standing characteristics (‘stand_characteristics_MG.tsv’) and soil chemistry (‘soil_chemistry_SE.tsv’ and ‘soil_chemistry_MG.tsv’) collected at each Malaise trap location, as well as data on biomass and specimen counts (biomass_count_IBA.tsv and biomass_count_SIIP.tsv).

Processed ASV data include ASV files after taxonomic annotation with SINTAX and phylogenetic reassignment (‘asv_taxonomy.tsv’), sequences in FASTA format based on the representative ASV (see above) for each cluster (‘cluster_reps.fasta’), ASV cluster designations and taxonomy (‘cluster_taxonomy.tsv’), consensus taxonomy of clusters (‘cluster_consensus_taxonomy.tsv’) and summed ASV counts for each cluster (‘cluster_counts.tsv’). These data are available at Figshare^61^. In the same repository we also provide taxonomy and count files for OTUs remaining after NEEAT filtering and cleaning to remove OTUs present in control samples.

We also provide an earlier upload of raw ASV data with corresponding metadata files in Figshare^62^. This upload contains ASVs from Malaise trap samples processed with mild lysis only, with the exception of 15 samples for which we also provide data from homogenates and preservative ethanol. These data were processed using an earlier version of the bioinformatic processing workflow, and we also provide the data resulting from this effort^63^. In this version, Swarm was run with setting ‘-d 13’, taxonomic re-assignment of ASVs using the insect phylogeny was not performed and removal of NUMTs and other potential noise was done using only taxonomic and abundance information. In summary, in this version of the workflow we removed ASVs present in more than 5% of blanks, ASVs unassigned at the family level, and clusters with less than 3 total counts summed across each dataset. For the Madagascar dataset, very few ASVs could be reliably assigned a taxonomy below the order level. Consequently, for the Madagascar dataset in this version we did not remove ASVs unassigned at the family level during the cleaning steps. In this version, homogenate samples from Sweden and soil and litter samples from both Sweden and Madagascar were not included, because they were unavailable at the time.

The data files provided contain ASVs that represent biological spike-ins. Specifically, the biological spike-ins used in Swedish datasets (lysates and homogenates) are *Gryllodes sigillatus* (Orthoptera: Gryllidae; in our datasets, the annotation of this taxon is *Gryllodes supplicans*; the reason is that this is the name used by GBIF for the matching CO1 sequences)*, Gryllus bimaculatus* (Orthoptera: Gryllidae), *Shelfordella lateralis* (Blattodea: Blattidae)*, Drosophila serrata, Drosophila bicornuta* and *Drosophila jambulina* (all Diptera: Drosophilidae; *D. jambulina* is annotated as ‘unclassified.Drosophila’ in our datasets due to name ambiguity in the annotation of the corresponding BIN in BOLD). The spike-ins used for Madagascar datasets (lysates) are *Orius majusculus* (Hemiptera: Anthocoridae), *Macrolophus pygmaeus* (Hemiptera: Miridae), *Delphastus pusillus* (Coleoptera: Coccinellidae), *Aphidius colemani* (Hymenoptera: Braconidae) *and Aphidoletes aphidimyza* (Diptera: Cecidomyiidae). Homogenate samples (only available from Sweden) also contain synthetic spike-ins (raw homogenate data only).

The ASVs corresponding to the OTUs that remained after NEEAT filtering and cleaning, together with their counts and metadata, can also be accessed and viewed interactively through the ASV-portal (https://asv-portal.biodiversitydata.se)^64^ at the Swedish Biodiversity Infrastructure (SBDI), as well as through the Global Biodiversity Information Facility (GBIF). In GBIF, the Swedish data can be assessed using 10.15468/veahzb for the lysate^65^, 10.15468/af5cwp for the ethanol^66^, 10.15468/awjycd for the homogenate^67^, and 10.15468/783jyb for the soil-litter dataset^68–70^, and the Madagascar data using 10.15468/6u5rum for the lysate and 10.15468/pad7pc for the litter dataset. Here all ASVs (and their counts) are present rather than just OTU-representative ASVs.

## Technical Validation

Throughout all steps of sample processing, we used negative control samples to control for cross-sample contamination. More specifically, we used negative controls during mild lysis and homogenization (‘buffer_blank’ and ‘buffer_blank_art_spikes’), DNA purification (‘extraction_neg’) and library preparation (‘pcr_neg’). Negative control samples can be identified in column ‘lab_sample_type’ in the ‘sequencing_metadata’ files available at Figshare^37^.

NUMTs and other types of noise were removed from the clustered ASV results using the NEEAT-filtering algorithm described above and we examined the median number of OTUs and sum of reads in negative controls and samples before and after this filtering step. For Swedish samples the median number of OTUs was 296 and 191, and the median sum of reads was 665k and 641k before and after filtering, respectively. For negative controls these numbers were 3 and 1 OTUs and 100 and 20 reads before and after filtering, respectively.

Samples in the Madagascar dataset had a median of 292 and 146 OTUs, and a median of 490k and 248k summed reads before and after filtering, respectively. Negative controls in this dataset had a median of 3 and 1 OTUs and 831 and 18 summed reads before and after filtering, respectively.

As part of data clean-up, it is usually advised to remove ASVs present in negative controls, or the maximum number of reads for those, from the entire dataset^71^. However, after careful inspection of our negative controls, we noticed that only a few ASVs were persistently showing up in control samples. The majority of ASVs seemed to be arthropod sequences that were present in the bulk samples, and also sporadically present in negative controls in relatively small numbers. This was presumably due to DNA spreading between samples through tiny droplets during sample processing, or to low-level of “index hopping”, leading to incorrect assignment of reads during sequencing, despite the use of double-unique indexes in library preparation^72^. Overall, this type of contamination was small and appeared random, leading us to the conclusion that removing all ASVs present in negative controls would impoverish the results without significantly improving quality. To ensure that we removed only true contaminants, we decided to filter out only those ASV clusters that showed up in more than 5% of negative controls that were successfully sequenced.

For Sweden, only seven clusters fit these criteria. Five of these were yeasts or bacteria; the remaining two were *Homo sapiens* (assigned to *H. neanderthalensis* due to GBIF removal of human data), and *Salticus scenicus* (zebra spider, a jumping spider). The latter is common in the building where samples were processed.

For Madagascar, 23 clusters fit the criteria. In addition to bacteria, yeasts, a plant (wheat or barley) and *Homo sapiens*, there were 14 arthropod clusters: *Allacma fusca*, *Entomobrya sp*., *Orchesella flavescens*, *O. cincta* and *Lepidocyrtus* sp. (Collembola); *Shelfordella lateralis* (Blattodea); *Nematopogon metaxella*, *Oligia* sp. (Lepidoptera); *Helina depuncta* and *Helina sp*., *Leptogaster cylindrica*, *Sciapus platypterus*, *Sericomyia* sp. and *Sylvicola stackelbergi* (Diptera). Only *Entomobrya* sp. and *H. depuncta* occurred in more than 10% of control samples (13% and 12%, respectively). All of these clusters correspond to common species or spike-ins in the Swedish samples, which were processed before the Madagascar samples in the same lab. Many of them also occur in or around the building where samples were processed.

Some ASVs were assigned to a reference sequence in the BOLD database annotated as *Zoarces gillii* (BOLD:AEB5125), a fish found between Japan and eastern Korea. Closer inspection revealed that this was a mis-annotated bacterial sequence and ASVs assigned to this reference most likely represent bacterial sequences in our dataset. ASVs assigned to this species (232 ASVs in 55 OTUs for Sweden and 99 ASVs in 34 OTUs for Madagascar) were consequently removed. This record has been deleted from BOLD after our custom reference database was constructed.

After removal of OTUs present in > 5% of negative controls, as well as the *Z. gillii* OTUs, the median number of OTUs and median sum of reads in negative controls for both Sweden and Madagascar were zero. Samples in Sweden had a median of 189 OTUs and 640k total read counts, respectively. For Madagascar, samples had a median of 145 OTUs and 248k total read counts (Table 2).

**Table 2:** Median number of OTUs and sum of read counts.

Dataset	Output type	Sample type	OTUs	Sum of read counts
Sweden	Swarm	Sample	296	665348
		Negative control	3	100
	NEEAT-filtered	Sample	191	640509
		Negative control	1	20
	Cleaned	Sample	189	640475
		Negative control	0	0
Madagascar	Swarm	Sample	292	490458
		Negative control	3	831
	NEEAT-filtered	Sample	146	247892
		Negative control	1	18
	Cleaned	Sample	145	247892
		Negative control	0	0

**Fig. 4:**
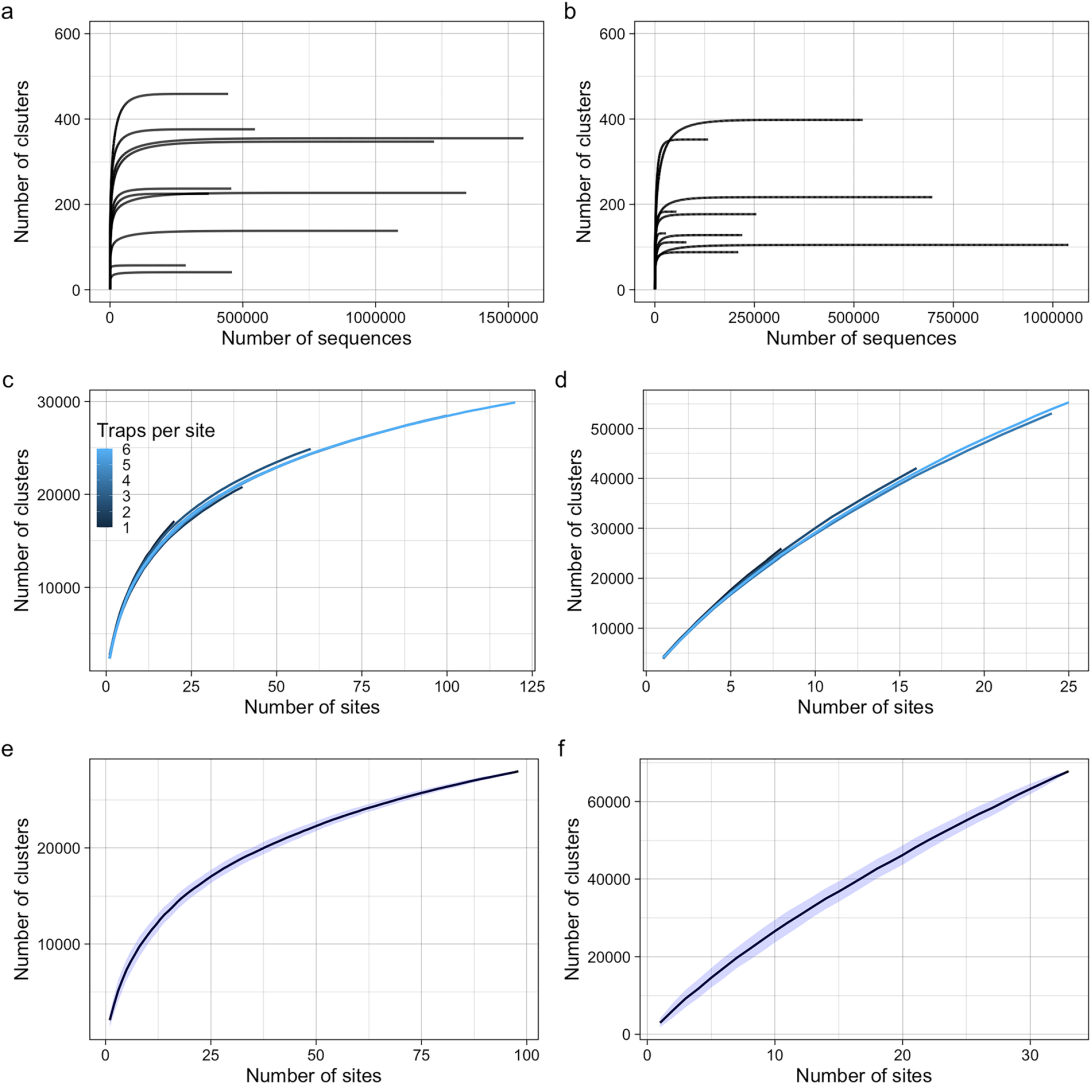
Results from the technical validation exercise to demonstrate sufficiency of the sequencing depth and the spatial sampling design to capture arthropod diversity in Sweden and Madagascar. Rarefaction curves illustrate cluster (species) accumulation with increasing sampling depth for 10 random samples in Sweden (**a**) and Madagascar (**b**). Accumulation of species with an increasing number of traps for Sweden (**c**) and Madagascar (**d**). Each individual line represents the average accumulation of species given a specified site-level survey effort, that is, traps per site (indicated by colour). The X-axis (total number of traps), represents the pooled trap number across all multi-trap sites. Accumulation of species with increasing spatial sampling effort (number of sites) for Sweden (**e**) and Madagascar (**f**).

To assess whether the sampling and sequencing design was sufficient to characterize insect diversity at different levels, we performed three separate analyses. We performed analyses to assess (i) whether sequencing depth was sufficient to detect most species present in an individual sample (ii) whether the number of traps per site was sufficient to characterize the site-level community, (iii) whether the number of sites was sufficient to characterize the fauna of the region (i.e., the entirety of Sweden, or all forests of Madagascar). To answer these questions, we used functionality from the ‘vegan’ R package^73^, specifically the ‘rarecurve’ (Fig. 4a,b) and ‘specaccum’ (Fig. 4c–f) functions.

To address the first question, we performed sample-level rarefaction on the sequencing depth of 10 random samples from both countries (Fig. 4a,b). This allowed us to examine whether the number of sequences was sufficient to detect all species within a sample. As all curves level off before 500,000 reads, the average of 1.24 million reads per sample (median = 1.09) was more than sufficient to detect the vast majority of species.

Within-site sampling efficiency was assessed using samples from sites that contained more than one trap (multitrap sites in Fig. 1). To achieve this, we iteratively increased the number of traps representing each site and constructed species accumulation curves from samples pooled across all sites. For each iteration, we randomly sampled 20 permutations of *N* traps from each site (N = 1:6 traps per site for Sweden, N = 1:4 traps per site for Madagascar). For example, at the first iteration (N = 1) a single permutation consisted of sampling one random trap per site - representing the lowest level of survey effort. In the second iteration (N = 2), a single permutation consisted of two random traps per site. When *N* was equal to the maximum number of site-level traps (i.e., we were using the full sample*)*, only one permutation was possible. For each permutation of traps, data was pooled and a species accumulation curve was constructed. Within each iteration (i.e., for each level of survey effort), the mean of the permutation-level curves was then taken to find the average species detection rate across sampling intensities. This analysis allows us to examine if, on average, more site-level sampling (i.e., traps) results in increased species detection. If lower within-site survey effort produces fewer species detections, we would expect lower intensities to produce less steep accumulation curves. Despite the close proximity of sampling locations at multi-trap sites and long sampling period of the survey, the trajectories of each species accumulation curve in panels 4 C and 4D demonstrate that, on average, more traps per location generally detected a considerably larger number of species, suggesting that even more detailed sampling at the site-level would be required to better characterise the annual invertebrate fauna in both countries.

Finally, at the country level, our analyses show that for Sweden, the total number of surveyed sites was bordering on sufficient to capture most species richness, that can be captured with Malaise traps, across the country, as the curve begins to level off at higher sampling site numbers (Fig. 4e). However, for Madagascar, the analysis suggests that 33 sites were still not sufficient to fully capture nation-wide species richness (Fig. 4f).

**Fig. 5:**
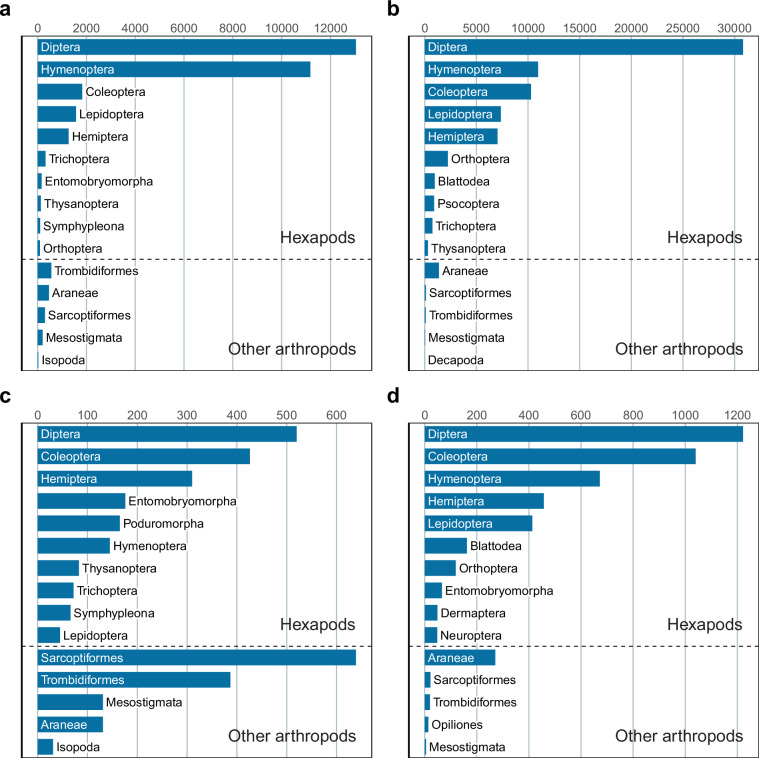
Order-level taxonomic composition of the IBA datasets (number of OTUs). (**a**) Swedish Malaise trap samples. (**b**) Malagasy Malaise trap samples. (**b**) Swedish soil and litter samples. (**d**) Malagasy litter samples. The bar plots show the top ten insect (Hexapoda) orders, and the top five other arthropod orders. Note that the Swedish datasets represent more intense sampling (four times as many samples) of a less diverse fauna than the Malagasy datasets. Also note that the Malagasy litter data does not include any soil samples, in contrast to the Swedish data.

**Fig. 6:**
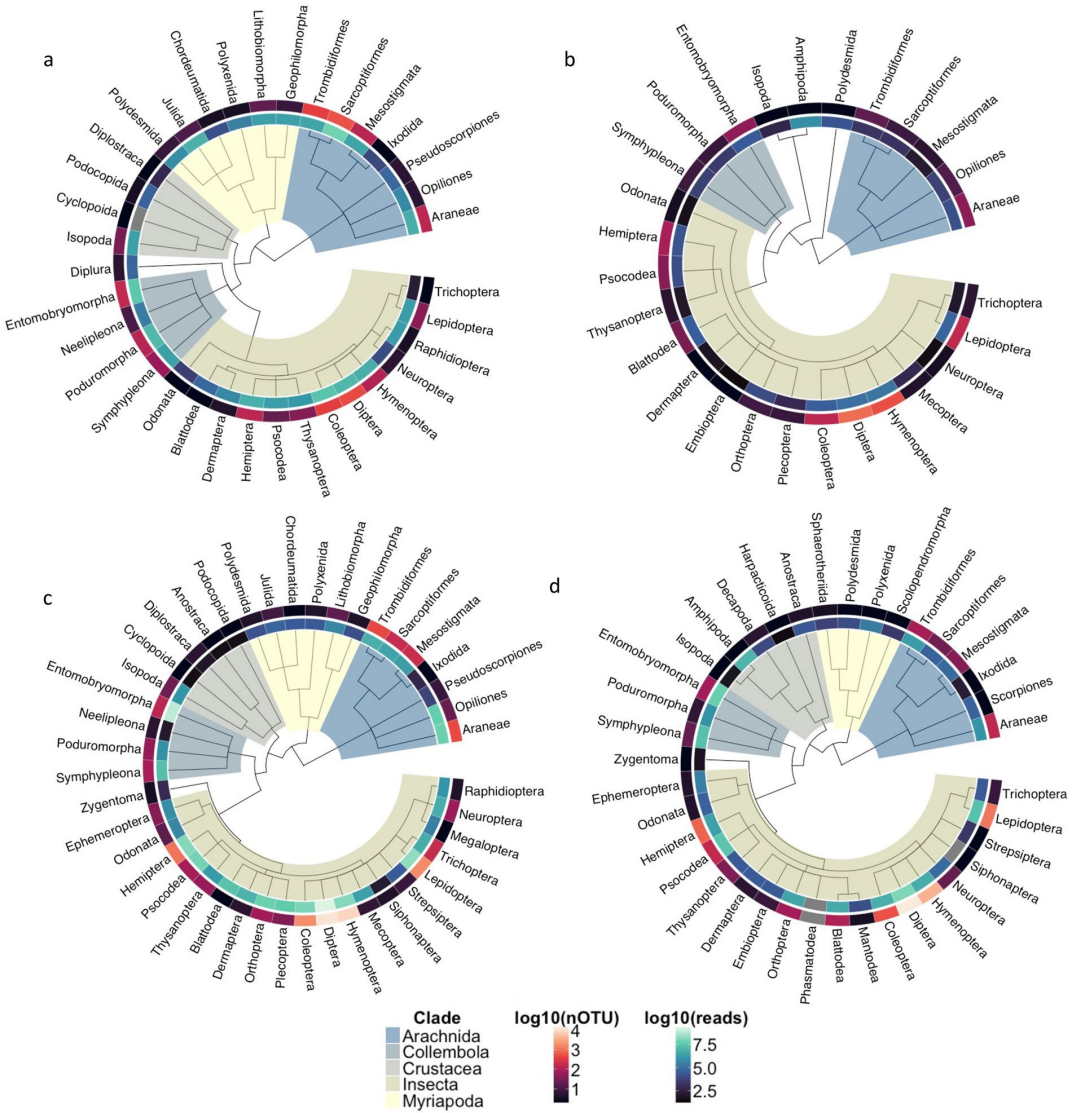
Order-level taxonomic composition of samples. Swedish (**a**) and Malagasy (**b**) Malaise trap samples, Swedish soil and litter samples (**c**), Malagasy litter samples (**d**). Displayed are the Arthropod taxonomic trees with each tip representing an order. Major clades are highlighted across this tree. The inner ring around each order represents the base 10 logarithm of the total number of reads for each order and the outer ring represents the base 10 logarithm of the total number of assigned OTUs. For clarity, only orders with at least 100 reads are displayed.

In terms of taxonomic composition, the Malaise trap datasets are distinctly different from the soil and litter samples (Figs. 5 and 6). Malaise trap samples are dominated by Diptera and Hymenoptera in both countries, even though the Diptera are more prominent and the Hymenoptera less so in Madagascar. These orders are followed by the remaining three major insect orders (Coleoptera, Lepidoptera, and Hemiptera). Other arthropods constitute a relatively small proportion of the diversity. In the soil and litter samples, mites and spiders constitute a larger fraction of the diversity, particularly in Sweden. Among the insects, the bristletails (Entomobryomorpha and Poduromorpha) are also considerably more prominent than in Malaise trap samples. These patterns largely agree with expectations and suggest that the data properly reflects the diversity of the sampled faunas^8,22,23,74^.

The precision and accuracy of the Swedish data are evaluated in^31^ as part of the methods development effort, and in more detail for Swedish Lepidoptera (butterflies and moths) in^75^. Thanks to a long tradition of naturalists and the relative completeness of the reference libraries, the Swedish insect fauna permits powerful tests of the quality of the data from the Insect Biome Atlas project.

The 1,705 Lepidoptera OTUs found in Swedish Malaise trap samples (using version 1 of the data processing pipeline on lysates only) matched 1,535 unique species previously known from Sweden and included a number of tentative new species records^75^. This suggests an over-splitting rate of less than 10%. The data covered 51% of the species ever recorded from the country, and 57% of the permanent resident species; the coverage was roughly the same across Lepidoptera families. Full-length barcoding of individual specimens representing 10 of the clusters that did not match known Swedish species confirmed that eight of them represented new species for the country or to science, or new and previously unknown CO1 variants at genetic distances that typically signal unique species.

Similar analyses across all families of Swedish insects also indicate good accuracy and precision of the processed IBA data^31^. After chimera removal, clustering with swarm had a precision of 0.98 and a recall of 0.91 when assessed against the species annotation of the ASVs. That is, the clusters were highly consistent with taxonomy, but there was a slight tendency towards over-splitting as shown by the lower recall values. A considerable amount of this over-splitting disappeared after noise removal with the NEEAT algorithm. In the cleaned data, the vast majority of well-known Swedish insect families are represented by fewer clusters than described species, with only a few instances apparently representing substantial over-splitting. Many families are represented by slightly more than half of the recorded number of species, suggesting that the quality of the data is similar to that of Lepidoptera. The over-splitting of Orthoptera, an order notorious for its rampant NUMTs, is considerably lower in the IBA data than in comparable datasets^76^.

## Usage Notes

**Fig. 7:**
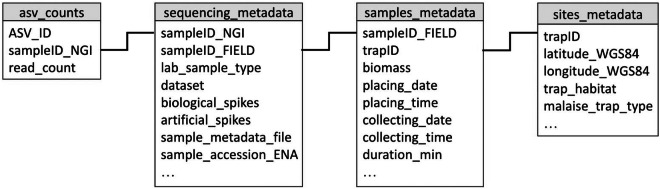
Simplified data model describing how the key fields in the main data and metadata files are linked.

The primers used for amplification were not removed from raw sequencing data and are therefore part of the reads deposited at the ENA^60^. The biological and synthetic spike-in sequences are also part of the raw data deposited at ENA. The clusters corresponding to spike-ins were not removed during the bioinformatic processing, so they are also included in the cleaned cluster data. The relationships between the metadata and ASV files are visualized in the simplified data model shown in Fig. 7. The sample names used in the ASV and clustered files refer to the ‘sampleID_NGI’ field in the corresponding ‘sequencing_metadata’ file for each country (‘CO1_sequencing_metadata_SE.tsv’ and ‘CO1_sequencing_metadata_MG.tsv’). To retrieve metadata information about the original sample from which ASV data were obtained, you need to use the corresponding ‘sequencing_metadata’ file to identify the ‘sampleID_FIELD’ associated with the ‘sampleID_NGI’ that the ASV data refers to. Once you have the ‘sampleID_FIELD’ information, you can then use column ‘sample_metadata_file’ to identify the ‘samples_metadata’ file where all metadata associated with that specific sample (e.g., ‘placing_date’, ‘placing_time’, ‘biomass’, ‘trapID’, etc) is stored. From the ‘sites_metadata’ files you can retrieve all information associated with the sampling sites (e.g., ‘latitudeWGS84’, ‘longitudeWGS84’, ‘trap_habitat’). To identify the location of each sample, use the “trapID” column from the corresponding ‘sample_metadata’ file.

## Supplementary information

Dataset 1

## Data Availability

Code for generating the statistics and plotting the figures presented in this paper are available at GitHub (https://github.com/insect-biome-atlas/paper-data/tree/main/code). To facilitate usage of the data, we provide a set of scripts to process the data further, including scripts to remove spike-in reads and control samples, to use spike-in data (biological and synthetic) and weight data to generate calibrated read numbers and to generate distribution maps for Sweden or Madagascar based on site occurrence data. These scripts are available at https://github.com/insect-biome-atlas/utils.

## References

[1] Sverdrup-Thygeson, A. *Extraordinary Insects: Weird, Wonderful, Indispensable: The Ones Who Run the World*. UK edition. Mudlark (2019).

[2] Crespo-Pérez, V., Kazakou, E., Roubik, D. W. & Cárdenas, R. E. The importance of insects on land and in water: a tropical view. *Vectors Med Vet Entomol • Spec Sect Insects UN Sustain Dev Goals* **40**, 31–38, 10.1016/j.cois.2020.05.016 (2020). 10.1016/j.cois.2020.05.016 32563991

[3] Grames, E. M., Montgomery, G. A., Youngflesh, C., Tingley, M. W. & Elphick, C. S. The effect of insect food availability on songbird reproductive success and chick body condition: Evidence from a systematic review and meta-analysis. *Ecol Lett* **26**(4), 658–673, 10.1111/ele.14178 (2023). 36798988 10.1111/ele.14178

[4] Kagata, H. & Ohgushi, T. Bottom-up trophic cascades and material transfer in terrestrial food webs. *Ecol Res* **21**(1), 26–34, 10.1007/s11284-005-0124-z (2006).

[5] Yang, L. H. & Gratton, C. Insects as drivers of ecosystem processes. *Ecology.* **2**, 26–32, 10.1016/j.cois.2014.06.004 (2014). 10.1016/j.cois.2014.06.004 32846721

[6] Mora, C., Tittensor, D. P., Adl, S., Simpson, A. G. B. & Worm, B. How Many Species Are There on Earth and in the Ocean? *PLoS Biol.* **9**(8), e1001127, 10.1371/journal.pbio.1001127 (2011). 21886479 10.1371/journal.pbio.1001127 PMC3160336

[7] Stork, N. E. How Many Species of Insects and Other Terrestrial Arthropods Are There on Earth? *Annu Rev Entomol.* **63**(1), 31–45, 10.1146/annurev-ento-020117-043348 (2018). 28938083 10.1146/annurev-ento-020117-043348

[8] Srivathsan, A. *et al*. Convergence of dominance and neglect in flying insect diversity. *Nat Ecol Evol.* **7**(7), 1012–1021, 10.1038/s41559-023-02066-0 (2023). 37202502 10.1038/s41559-023-02066-0 PMC10333119

[9] Hallmann, C. A. *et al*. More than 75 percent decline over 27 years in total flying insect biomass in protected areas. *PLOS ONE* **12**(10), e0185809, 10.1371/journal.pone.0185809 (2017). 29045418 10.1371/journal.pone.0185809 PMC5646769

[10] Lister, B. C. & Garcia, A. Climate-driven declines in arthropod abundance restructure a rainforest food web. *Proc Natl Acad Sci* **115**(44), E10397–E10406, 10.1073/pnas.1722477115 (2018). 30322922 10.1073/pnas.1722477115 PMC6217376

[11] Møller, A. P. Parallel declines in abundance of insects and insectivorous birds in Denmark over 22 years. *Ecol Evol.* **9**(11), 6581–6587, 10.1002/ece3.5236 (2019). 31236245 10.1002/ece3.5236 PMC6580276

[12] Outhwaite, C. L., McCann, P. & Newbold, T. Agriculture and climate change are reshaping insect biodiversity worldwide. *Nature* **605**(7908), 97–102, 10.1038/s41586-022-04644-x (2022). 35444282 10.1038/s41586-022-04644-x

[13] Seibold, S. *et al*. Arthropod decline in grasslands and forests is associated with landscape-level drivers. *Nature* **574**(7780), 671–674, 10.1038/s41586-019-1684-3 (2019). 31666721 10.1038/s41586-019-1684-3

[14] Thomas, C. D., Jones, T. H. & Hartley, S. E. Insectageddon”: A call for more robust data and rigorous analyses. *Glob Change Biol.* **25**(6), 1891–1892, 10.1111/gcb.14608 (2019). 10.1111/gcb.14608 30821400

[15] van Klink, R. *et al*. Disproportionate declines of formerly abundant species underlie insect loss. *Nature*. 10.1038/s41586-023-06861-4 (2023). 10.1038/s41586-023-06861-4 PMC11006610 38123681

[16] van Klink, R. *et al*. Meta-analysis reveals declines in terrestrial but increases in freshwater insect abundances. *Science* **368**(6489), 417–420, 10.1126/science.aax9931 (2020). 32327596 10.1126/science.aax9931

[17] Basset, Y. & Lamarre, G. P. A. Toward a world that values insects. *Science* **364**(6447), 1230–1231, 10.1126/science.aaw7071 (2019). 31249044 10.1126/science.aaw7071

[18] Cardoso, P. *et al*. Scientists’ warning to humanity on insect extinctions. *Biol Conserv* **242**, 108426, 10.1016/j.biocon.2020.108426 (2020).

[19] Harvey, J. A. *et al*. Scientists’ warning on climate change and insects. *Ecol Monogr* **93**(1), e1553, 10.1002/ecm.1553 (2023).

[20] Harvey, J. A. *et al*. International scientists formulate a roadmap for insect conservation and recovery. *Nat Ecol Evol* **4**(2), 174–176, 10.1038/s41559-019-1079-8 (2020). 31907382 10.1038/s41559-019-1079-8

[21] Wilson, R. J. & Fox, R. Insect responses to global change offer signposts for biodiversity and conservation. *Ecol Entomol* **46**(4), 699–717, 10.1111/een.12970 (2021).

[22] Karlsson, D., Hartop, E., Forshage, M., Jaschhof, M. & Ronquist, F. The Swedish Malaise Trap Project: A 15 Year Retrospective on a Countrywide Insect Inventory. *Biodivers Data J* **8**, e47255, 10.3897/BDJ.8.e47255 (2020). 32015667 10.3897/BDJ.8.e47255 PMC6987249

[23] Ronquist, F. *et al*. Completing Linnaeus’s inventory of the Swedish insect fauna: Only 5,000 species left? *PLOS ONE* **15**(3), e0228561, 10.1371/journal.pone.0228561 (2020). 32130216 10.1371/journal.pone.0228561 PMC7055846

[24] van Klink, R. *et al*. Emerging technologies revolutionise insect ecology and monitoring. *Trends Ecol Evol* **37**(10), 872–885, 10.1016/j.tree.2022.06.001 (2022). 35811172 10.1016/j.tree.2022.06.001

[25] Hewitt, G. M. Post-glacial re-colonization of European biota. *Biol J Linn Soc* **68**(1), 87–112, 10.1006/bijl.1999.0332 (1999).

[26] Antonelli, A. *et al*. Madagascar’s extraordinary biodiversity: Evolution, distribution, and use. *Science* **378**(6623), eabf0869, 10.1126/science.abf0869 (2022). 36454829 10.1126/science.abf0869

[27] Ståhl, G. *et al*. National Inventory of Landscapes in Sweden (NILS)—scope, design, and experiences from establishing a multiscale biodiversity monitoring system. *Environ Monit Assess* **173**(1), 579–595, 10.1007/s10661-010-1406-7 (2011). 20237838 10.1007/s10661-010-1406-7

[28] Iwaszkiewicz-Eggebrecht, E. *et al*. FAVIS: Fast and versatile protocol for non-destructive metabarcoding of bulk insect samples. *PloS One* **18**(7), e0286272, 10.1371/journal.pone.0286272 (2023). 37467453 10.1371/journal.pone.0286272 PMC10356154

[29] Iwaszkiewicz-Eggebrecht, E. *et al*. FAVIS: Fast and Versatile protocol for metabarcoding of bulk Insect Samples from large-scale diversity monitoring projects v2. *protocols.io*. 10.17504/protocols.io.kqdg36261g25/v2 (2023). 10.1371/journal.pone.0286272 PMC10356154 37467453

[30] Persson, N., Johansson, H., Iwaszkiewicz-Eggebrecht, E. & Miraldo, A. Sample homogenization and DNA extraction for bulk insect catches. *protocols.io*. 10.17504/protocols.io.yxmvm31bbl3p/v1(2024)

[31] Sundh, J. *et al*. HAPP: High-Accuracy Pipeline for Processing of deep metabarcoding data. (2024) 10.1371/journal.pcbi.1013558 PMC12622834 41202092

[32] van Dijk, L. J. A. *et al*. Biotic and abiotic drivers of ecosystem functioning differ between a temperate and a tropical region. 10.1101/2024.02.28.582312(2024)

[33] van Dijk, L. J. A. *et al*. Temperature and water availability drive insect seasonality across a temperate and a tropical region. *Proceedings of the Royal Society B*. **291**(20240090), 10.1098/rspb.2024.0090 (2024) 10.1098/rspb.2024.0090 PMC11285739 38889793

[34] Ratnasingham, S., Hebert P. D. N. bold: The Barcode of Life Data System (http://www.barcodinglife.org). *Mol Ecol Notes.* 7(3), 355-364, 10.1111/j.1471-8286.2007.01678.x (2024) 10.1111/j.1471-8286.2007.01678.x PMC1890991 18784790

[35] Tullgren, A. Ein sehr einfacher Ausleseapparat für terricole Tierformen. *Z Für Angew Entomol* **4**(1), 149–150, 10.1111/j.1439-0418.1918.tb00820.x (1918).

[36] Besuchet, C., Burckhardt, D. H. & Löbl, I. The “Winkler/Moczarski” Eclector as an Efficient Extractor for Fungus and Litter Coleoptera. *The Coleopterists Bulletin* **41**(4), 392–394 (1987).

[37] Miraldo, A. *et al*. Amplicon sequence variants from the Insect Biome Atlas project. 10.17044/scilifelab.25480681.v5 (2024).

[38] Iwaszkiewicz-Eggebrecht, E., Prus-Frankowska, M. & Łukasik, P. Synthetic COI spike-ins for use in metabarcoding-based insect biodiversity surveys. *protocols.io*. 10.17504/protocols.io.14egn33ryl5d/v2 (2023).

[39] Arribas, P., Andújar, C., Salces-Castellano, A., Emerson, B. C. & Vogler, A. P. The limited spatial scale of dispersal in soil arthropods revealed with whole-community haplotype-level metabarcoding. *Mol Ecol* **30**(1), 48–61, 10.1111/mec.15591 (2021). 32772446 10.1111/mec.15591

[40] Arribas, P., Andújar, C., Hopkins, K., Shepherd, M. & Vogler, A. P. Metabarcoding and mitochondrial metagenomics of endogean arthropods to unveil the mesofauna of the soil. *Methods Ecol Evol* **7**(9), 1071–1081, 10.1111/2041-210X.12557 (2016).

[41] Glenn, T. C. *et al*. Adapterama I: universal stubs and primers for 384 unique dual-indexed or 147,456 combinatorially-indexed Illumina libraries (iTru & iNext). Lazo G, ed. *PeerJ* **7**, e7755, 10.7717/peerj.7755 (2019). 31616586 10.7717/peerj.7755 PMC6791352

[42] Elbrecht, V. *et al*. Validation of COI metabarcoding primers for terrestrial arthropods. *Pochon X, ed. PeerJ* **7**, e7745, 10.7717/peerj.7745 (2019). 10.7717/peerj.7745 PMC6786254 31608170

[43] Elbrecht, V. & Leese, F. Validation and Development of COI Metabarcoding Primers for Freshwater Macroinvertebrate Bioassessment. *Front Environ Sci*. **5**, https://www.frontiersin.org/articles/10.3389/fenvs.2017.00011 (2017).

[44] Bonath F. *Increased Complexity of Amplicon Libraries Using Phased Primers*. National Genomics Infrastructure; 2021.

[45] Wu, L. *et al*. Phasing amplicon sequencing on Illumina Miseq for robust environmental microbial community analysis. *BMC Microbiol* **15**(1), 125, 10.1186/s12866-015-0450-4 (2015). 26084274 10.1186/s12866-015-0450-4 PMC4472414

[46] Glenn, T. C. *et al*. Adapterama II: universal amplicon sequencing on Illumina platforms (TaggiMatrix). *PeerJ* **7**, e7786, 10.7717/peerj.7786 (2019). 31616589 10.7717/peerj.7786 PMC6791344

[47] Martin, M. Cutadapt removes adapter sequences from high-throughput sequencing reads. *EMBnetjournal Vol 17 No 1 Gener Seq Data Anal*. 10.14806/ej.17.1.200 (2011).

[48] Straub, D. *et al*. Interpretations of Environmental Microbial Community Studies Are Biased by the Selected 16S rRNA (Gene) Amplicon Sequencing Pipeline. *Front Microbiol*. **11**, https://www.frontiersin.org/journals/microbiology/articles/10.3389/fmicb.2020.550420 (2020). 10.3389/fmicb.2020.550420 PMC7645116 33193131

[49] Callahan, B. J. *et al*. DADA2: High-resolution sample inference from Illumina amplicon data. *Nat Methods* **13**(7), 581–583, 10.1038/nmeth.3869 (2016). 27214047 10.1038/nmeth.3869 PMC4927377

[50] Bohmann, K. *et al*. Strategies for sample labelling and library preparation in DNA metabarcoding studies. *Mol Ecol Resour* **22**(4), 1231–1246, 10.1111/1755-0998.13512 (2022). 34551203 10.1111/1755-0998.13512 PMC9293284

[51] Kircher, M., Sawyer, S. & Meyer, M. Double indexing overcomes inaccuracies in multiplex sequencing on the Illumina platform. *Nucleic Acids Res* **40**(1), e3–e3, 10.1093/nar/gkr771 (2012). 22021376 10.1093/nar/gkr771 PMC3245947

[52] Edgar, R. C. SINTAX: a simple non-Bayesian taxonomy classifier for 16S and ITS sequences. *bioRxiv*. 10.1101/074161 (2016).

[53] Rognes, T., Flouri, T., Nichols, B., Quince, C. & Mahé, F. VSEARCH: a versatile open source tool for metagenomics. *Hrbek T, ed. PeerJ* **4**, e2584, 10.7717/peerj.2584 (2016). 10.7717/peerj.2584 PMC5075697 27781170

[54] Sundh, J. COI reference sequences from BOLD DB. SciLifeLab. 10.17044/scilifelab.20514192.v4 (2022).

[55] Barbera, P. *et al*. EPA-ng: Massively Parallel Evolutionary Placement of Genetic Sequences. *Syst Biol* **68**(2), 365–369, 10.1093/sysbio/syy054 (2019). 30165689 10.1093/sysbio/syy054 PMC6368480

[56] Chesters, D. Construction of a Species-Level Tree of Life for the Insects and Utility in Taxonomic Profiling. *Syst Biol* **66**(3), 426–439, 10.1093/sysbio/syw099 (2017). 27798407 10.1093/sysbio/syw099 PMC5837528

[57] Czech, L., Barbera, P. & Stamatakis, A. Genesis and Gappa: processing, analyzing and visualizing phylogenetic (placement) data. *Bioinformatics* **36**(10), 3263–3265, 10.1093/bioinformatics/btaa070 (2020). 32016344 10.1093/bioinformatics/btaa070 PMC7214027

[58] Edgar, R. C., Haas, B. J., Clemente, J. C., Quince, C. & Knight, R. UCHIME improves sensitivity and speed of chimera detection. *Bioinformatics* **27**(16), 2194–2200, 10.1093/bioinformatics/btr381 (2011). 21700674 10.1093/bioinformatics/btr381 PMC3150044

[59] Mahé, F., Rognes, T., Quince, C., de Vargas, C. & Dunthorn, M. Swarm: robust and fast clustering method for amplicon-based studies. *Cohan F, ed. PeerJ* **2**, e593, 10.7717/peerj.593 (2014). 10.7717/peerj.593 PMC4178461 25276506

[60] *ENA European Nucleotide Archive* https://identifiers.org/ena.embl:PRJEB61109 (2025).

[61] Miraldo, A. *et al*. Processed ASV data from the Insect Biome Atlas Project. 10.17044/scilifelab.27202368.v3 (2024).

[62] Miraldo, A. *et al*. Amplicon sequence variants from the Insect Biome Atlas project. 10.17044/scilifelab.25480681.v1 (2024)

[63] Miraldo, A. *et al*. Processed ASV data from the Insect Biome Atlas Project. 10.17044/scilifelab.27202368.v1 (2024).

[64] Prager, M., Lundin, D., Ronquist, F. & Andersson, A. F. ASV portal: an interface to DNA-based biodiversity data in the Living Atlas. *BMC Bioinformatics* **24**(1), 6, 10.1186/s12859-022-05120-z (2023). 36604610 10.1186/s12859-022-05120-z PMC9817246

[65] Miraldo, A. *et al*. CO1 Amplicon Sequence Variants of bulk arthropod samples (mild lysis) collected with Malaise traps from the Insect Biome Atlas project in Sweden. 10.15468/veahzb (2024).

[66] Miraldo, A. *et al*. CO1 Amplicon Sequence Variants of bulk arthropod samples (preservative ethanol) collected with Malaise traps from the Insect Biome Atlas project in Sweden. Version 1.1. 10.15468/af5cwp (2024).

[67] Miraldo, A. *et al*. CO1 Amplicon Sequence Variants of bulk arthropod samples (homogenized) collected with Malaise traps from the Insect Biome Atlas project in Sweden. 10.15468/awjycd (2024).

[68] Miraldo, A. *et al*. CO1 Amplicon Sequence Variants of bulk arthropod samples (mild lysis) collected with Malaise traps from the Insect Biome Atlas project in Madagascar (2025).

[69] Miraldo, A. *et al*. CO1 Amplicon Sequence Variants of leaf litter arthropod communities collected at Malaise traps from the Insect Biome Atlas project in Madagascar (2025).

[70] Miraldo, A. *et al*. CO1 Amplicon Sequence Variants of soil and leaf litter arthropod communities collected at Malaise traps from the Insect Biome Atlas project in Sweden. Version 1.1. 10.15468/783jyb (2024).

[71] Alberdi, A., Aizpurua, O., Gilbert, M. T. P. & Bohmann, K. Scrutinizing key steps for reliable metabarcoding of environmental samples. *Methods Ecol Evol* **9**(1), 134–147, 10.1111/2041-210X.12849 (2018).

[72] MacConaill, L. E. *et al*. Unique, dual-indexed sequencing adapters with UMIs effectively eliminate index cross-talk and significantly improve sensitivity of massively parallel sequencing. *BMC Genomics* **19**(1), 30, 10.1186/s12864-017-4428-5 (2018). 29310587 10.1186/s12864-017-4428-5 PMC5759201

[73] Oksanen, J. *et al*. vegan: Community Ecology Package. Accessed May 24, 2024 https://cran.r-project.org/web/packages/vegan/index.html (2024).

[74] Coleman, D. & Callaham, M. C. Jr. *Fundamentals of Soil Ecology: Third Edition*. 2017.

[75] Iwaszkiewicz-Eggebrecht, E. *et al*. High-throughput biodiversity surveying sheds new light on the brightest of insect taxa. *bioRxiv*. 10.1101/2024.10.25.620209 (2024). 10.1098/rspb.2024.2974 PMC12074807 40359979

[76] Buchner, D. *et al*. Upscaling biodiversity monitoring: Metabarcoding estimates 31,846 insect species from Malaise traps across Germany. *Mol Ecol Resour* **25**(1), e14023, 10.1111/1755-0998.14023 (2025). 39364584 10.1111/1755-0998.14023 PMC11646302

